# Medical History from the Earliest Times

**Published:** 1893-01-14

**Authors:** E. T. Withington


### MEDICAL HISTORY FROM THE EARLIEST TIMES.

By E. T. WITHINGTON, M.A., M.B. Oxon.

### XXXVIII.—THE ARABO-SCHOLASTIC REVIVAL. (1) MEDICINE.

THE thirteenth century, like the age of Pericles and the Renaissance, is one of the great epochs of human history, and has been well characterised as "the trumpet call which summoned the Middle Ages into the modern world." Able and far-sighted politicians inaugurated important developments in Church and State, while theology, philosophy, and science were furthered by the labours of "great," "wonderful," "subtle," "universal," and "irrefragable" doctors. The impulse to this new activity, especially in science, was derived largely from the Arabs, and above all from the study of the physical and metaphysical works of Aristotle, which were translated first from the Arabic and afterwards from the Greek. Their effect was to revolutionise the dominant Christian view of Nature, hitherto looked upon as a ruin, beset with pitfalls and haunted by demons, into whose clutches the rash explorer of its mysteries was certain to fall, if he had not already made an unholy compact with their Master, the prince of this world. This was now replaced, at least in part, by the Greek view of the universe as a harmonious whole, "the diapason closing full in man," who himself forms a little universe or microcosm.

An important agent and example of this change was Albertus Magnus, who was at once declared "Blessed" by the Church, and a sorcerer by the general voice, and whose excessive praises—"great in magic, greater in philosophy, greatest in theology," "the Christian Aristotle, "*major Platone, vix inferior Salomone*—were perhaps partly due to the fact that here at length was a man of science, approved by theologians, who might therefore be safely eulogised. But we must pass over the Blessed Albert, and even the "wonderful doctor," Roger Bacon, whose achievements may be considered, at least by his countrymen, more important than those of the "Doctor Magnus," and confine ourselves to physicians in the narrower sense of the word.

Two men stand out pre-eminent as types of the Arabo-scholastic medicine of the thirteenth century, and their work will here be sketched as briefly as possible. Three nations, and nearly twenty towns, have disputed the honour of producing Arnald of Villanova 1235-1312), but an impartial foreigner may believe that he was born in Spain, educated chiefly in France, and that one of the works attributed to him was probably written by an Italian, Arnold of Naples or Novicomo. He tells us that he was doctor in the four faculties—theology, law, philosophy, and medicine, and he knew what were then the three learned languages—Arabic, Greek, and Hebrew. His theological works have perished in the flames, but we know that he attacked the Friars with the energy of a Wycliffe, and that his enemies convicted him of fourteen deadly errors—the most interesting being "that works of mercy and medicine are more acceptable to God than the sacrifice of the altar"—and called upon the Pope to deal with the heretic according to the customs of the Church. Clement V., however, though he took an active part in torturing Templars, and confiscating their goods, wisely considered that a physician, who was also an alchemist and possible gold-maker, might be put to better uses. He profited by his medical skill, and encouraged his chemical investigations, and the date of Arnald's decease is fixed by an encyclical letter, in which the Pope informs the Bishops of Christendom of the death of that pious and learned physician, bids them search diligently for a certain medical work dedicated to himself, and threatens with his apostolic anathema anyone who shall conceal or destroy it. But other Popes soon arose who knew not Arnald; thirteen of his works were publicly burnt, and even his commentary on the Salernitan "Regimen Sanitatis" was placed on the list of forbidden books.

Arnald was the alchemist of the new medicine, but his aim was not so much the transmutation of metals as the discovery of an universal remedy or elixir of life. He thought that some approach to this was made by the "alcohol" of the Arabs, the "aqua vini" or "vitæ," whose virtues, he says, were already known to many, and to which he attributed a special power of restoring youth to the aged. He further pointed out its remarkable property of extracting the essences of herbs and roots, and thus laid the foundation for the "tinctures" of the modern pharmacopœia. But while adopting the chemical innovations of the Arabs, he carried on at the same time the best tradition of the Salernitan school, its fondness for dietetics and hygiene. This is seen in the above-mentioned commentary, and in many of his aphorisms, such as "The modest and wise physician will never hasten to pharmacy unless compelled by necessity." His treatises on various chronic disorders usually end with a chapter of general advice to those affected by them, which form the germ of the "consilia," so characteristic of the next epoch, and the following is a condensed abstract of his counsel to sufferers from palpitation (tremor cordis). After an excellent account of the causes, varieties, and medical treatment of the affection, he continues, "Whoever will be freed from this disorder let him carefully study and diligently observe this salutary teaching lest he fall into a worse state. Let him avoid all coarse meats, such as that of oxen, goats, horses, camels, and water fowl, rich fish, pastry, new or unleavened bread, old, moist, or salt cheese, &c.; in short, all that is indigestible and all excess. Let him also avoid impure air, and air too hot or cold, or strong winds, and, since we must always breathe, let him cool the air in the dog-days by sprinkling branches of willow, myrtle, or vine with cold water, and adorn his chamber therewith; also by the evaporation of camphor, rosewater, and other cooling odours; and in winter let him have a fire and use warm odours, myrrh, lign-aloes, laudanum (ladanon), calamint. Let him take moderate exercise before eating, and rest entirely after it, till the food has left the stomach, and then ride horses or gently trotting mules, avoiding rapid ascents or descents. Frequent combing of the hair is a great help, especially after sleep, for it assists the evaporation of the humours which ascend to the head. He may use scented baths in the usual manner. Let his sleep and waking be measured by Nature; he may sleep soon, but not immediately after eating, and should lie first on the left side that the food may descend to the fundus of the stomach, and then on the right that it may pass on." Then follows a list of the things he may eat, in which we are astonished to find pickled pork, "sed licet raro." In winter and early spring he may eat twice a day, in summer he should take three smaller meals, but must never eat until he has fully digested the last meal, for nothing is worse than to add indigestion to indigestion. Then he discusses the three forms of drink—wine, water, and syrups—of which the first is a valuable medicine and food, but should always be taken with, and not before or after other food; and finally, he exhorts the patient to occupy his mind as much as possible with pleasant and cheerful thoughts.

As Arnald is the first great representative of the school of Montpellier, so Peter of Abano (1250-1315, is the earliest ornament of the University of Padua) afterwards so fertile in great physicians. Like Arnald, he came into collision with the theologians, and was only saved, by an opportune death, from being burnt alive. For his heresies were worse than those of his Spanish colleague; his astrological theories seemed to leave no room for the action of free-will or Providence; he denied the existence of the devil, and he dared to suggest that the case of a patient who lay in a trance three days might help to explain some Christian miracles, including even the raising of Lazarus. "But he lied in his iniquity," says Thomas, General of the Augustinians, "and received the reward of his error, for I was present when his bones were burnt in the city of Padua for these and his other heresies." A later writer asserts that his body was concealed by his housekeeper, Marietta, so they could only burn him in effigy; but which is correct matters little either to him or to us. The sentence of condemnation was publicly read, his memory was devoted to eternal infamy, and his wealth, which was great, to the use of the Church.

Peter of Abano represents the scholastic side of the new medicine, with its love of subtle distinctions and wordy dialectics, and he gained the title of "Conciliator" from his great work, "Conciliator Differentiarum," intended to reconcile the doctrines of physicians and philosophers. In it he discusses many curious and some important questions — whether fire is hot; whether pain is felt as pain; whether the head was meant for the brain or the eyes; how often we should eat in the day; whether we should sleep on the right or left side, &c. But we also find the important assertions that the veins originate from the heart and not from the liver, and that the brain is the only real seat of sensation, besides other discoveries which had to be rediscovered, such as the correct explanation of the rainbow, and the fact that the air has weight. But Peter was no less famous as a practitioner than as a disputant, and, in spite of his high fees, was in great request at Padua. As an example of this practical side, we may take his directions for the treatment of stomach-ache (de dolore stomachi). Narcotics, he says, should not be used except in the severest cases as a lesser evil, for they only produce an apparent cure. The indications are: "(1) To invoke the Giver of salvation, of the abundance of His mercy, to grant health to the sufferer, and to add to these prayers medicines endowed with Divine virtues, and the rules of life I have laid down in my treatise on migraine; (2) to immediately meet any vehement or serious symptoms; (3) to soothe the stomach and strengthen its digestive power; (4) to get rid of the *materia laedens*; (5) to treat any other organ which may be involved, and to be especially suspicious of the liver." He then pauses to ask, "What is pain?" and finally concludes that it is *rei contrariae contrarietatem sentire.* Food and drink should be avoided altogether, or should be of the lightest kind. Their nature he leaves to the physician, who must consider the patient's temperament, and which humour is in excess. Vomiting is not to be treated unless very severe, for it is a natural cure, and the stomach's shape and position have been specially ordained to enable it to get rid of excessive or injurious food in this way. If the pain continues, a warm bath may be given, and the patient should take warm aromatic wine with a little rosewater. Warmth, he declares, always tends to lessen pain, and proceeds to discuss the reasons. Finally comes the usual long list of drugs, the action of which he attempts to explain, but adds: "The drugs which cure this disease will increase the virulence of another very like it; men also are diverse, and subject to diverse operation of the same medicine. Who is sufficient for all these things? Pray, therefore, that God, the Giver of health, will direct you to the choice of the proper remedies."

